# Epidemiological, Clinical and Genetic Study of Hypophosphatasia in A Spanish Population: Identification of Two Novel Mutations in The Alpl Gene

**DOI:** 10.1038/s41598-019-46004-2

**Published:** 2019-07-02

**Authors:** Cristina García-Fontana, Juan M. Villa-Suárez, Francisco Andújar-Vera, Sheila González-Salvatierra, Gonzalo Martínez-Navajas, Pedro J. Real, José M. Gómez Vida, Tomás de Haro, Beatriz García-Fontana, Manuel Muñoz-Torres

**Affiliations:** 1 University Hospital San Cecilio, Instituto de Investigación Biosanitaria (Ibs.GRANADA), Granada, Spain; 2 Fundación para la Investigación Biosanitaria de Andalucía Oriental (FIBAO), Granada, Spain; 3 Clinical Analysis Unit, University Hospital San Cecilio, Granada, Spain; 4 Department of Medicine, University of Granada, Granada, Spain; 5 Gene Regulation, Stem Cells & Development Lab, GENYO, Centre for Genomics and Oncological Research: Pfizer-University of Granada-Andalusian Regional Government, Granada, Spain; 6 Department of Biochemistry and Molecular Biology I, University of Granada, Granada, Spain; 7 Pediatric Unit, University Hospital San Cecilio, Granada, Spain; 8 CIBERFES, Instituto de Salud Carlos III, Granada, Spain; 9 Endocrinology and Nutrition Unit, University Hospital San Cecilio, Granada, Spain

**Keywords:** Calcium and phosphate metabolic disorders, DNA sequencing

## Abstract

Hypophosphatasia (HPP) is a genetic disease caused by one or several mutations in ALPL gene encoding the tissue-nonspecific alkaline phosphatase affecting the mineralization process. Due to its low prevalence and lack of recognition, this metabolic disorder is generally confused with other more frequent bone disorders. An assessment of serum total alkaline phosphatase (ALP) levels was performed in 78,590 subjects. Pyridoxal-5′-phosphate (PLP) concentrations were determined and ALPL gene was sequenced in patients potentially affected by HPP. Functional validation of the novel mutations found was performed using a cell-based assay. Our results showed persistently low serum ALP levels in 0.12% of subjects. Among the studied subjects, 40% presented with HPP-related symptoms. Nine of them (~28%) had a history of fractures, 5 (~16%) subjects showed chondrocalcinosis and 4 (~13%) subjects presented with dental abnormalities. Eleven subjects showed increased PLP concentrations. Seven of them showed ALPL gene mutations (2 of the mutations corresponded to novel genetic variants). In summary, we identified two novel ALPL gene mutations associated with adult HPP. Using this protocol, almost half of the studied patients were diagnosed with HPP. Based on these results, the estimated prevalence of mild HPP in Spain could be up to double than previously reported.

## Introduction

Hypophosphatasia (HPP) is a rare disorder. The estimated prevalence of HPP in the European population is between 1/100,000 and 1/300,000 for severe forms and 1/6,370 for moderate forms^1^. However, no epidemiological studies on the prevalence of HPP in the Spanish population are available to date. The HPP is caused by one or several mutations in the ALPL gene encoding for the tissue-nonspecific alkaline phosphatase protein (TNSALP). This enzyme is involved in the dephosphorylation of a wide range of substrates, including inorganic pyrophosphate (PPi), phosphatidylethanolamine (PEA) and pyridoxal-5′-phosphate (PLP). A total of 388 mutations associated with HPP have been described to date^2^. The variety of mutations results in a highly variable clinical manifestations of HPP. Mild HPP is caused by a functional loss of one of the copies of the ALPL gene, leading to autosomal dominant inheritance, whereas, both alleles of the ALPL gene are affected in severe HPP^3^. The severe form of this disorder could be explained by autosomal recessive inheritance or by a dominant negative effect on an autosomal dominant mutation, where the gene product from one allele interferes with the monomer-monomer interaction^4^. The degree of hypophosphatasemia and TNSALP substrate accumulation reflect the severity of HPP^5^. Low levels of serum total alkaline phosphatase (ALP), in conjunction with the accumulation of natural substrates from TNSALP and with the clinical and radiographic findings are the hallmark for the diagnosis of HPP.

This disorder has been categorized in seven clinical forms including perinatal, infantile, childhood, adult, odonto-HPP, pseudo-HPP and benign prenatal HPP. The clinical manifestations of HPP can vary from severe (mainly in perinatal and infantile HPP) to moderate or mild (in adults)^6^.

The HPP in adults is typically detected during middle age^7–9^. The clinical manifestation of HPP in adults include loss of mineralization leading to recurrent metatarsal stress fractures, femoral pseudofractures (usually showing a delayed consolidation), history of rickets in childhood, musculoskeletal and joint pain^6^. This hypomineralization is caused by PPi accumulation in the extracellular matrix, which inhibits the formation of hydroxyapatite crystals^10^. This hypomineralization affects also the acellular cementum that covers the tooth root. For this reason, premature loss of deciduous or permanent dentition is another clinical manifestation of HPP^11^. Moreover, the increase of endogenous levels of PPi produce calcium pyrophosphate dihydrate crystal deposits in articular cartilage causing PPi arthropathy (including pseudogout), chondrocalcinosis and enthesopathy^12–14^ leading to musculoskeletal pain in HPP adult patients^6^.

In the pediatric population, clinical manifestations are more severe than in adults, being perinatal HPP the most severe. This form is manifested *in utero* with profound skeletal hypomineralization^10^, usually causing death after few days of life, due to defects in thorax and hypoplastic lungs leading to asphyxia^15^. In infantile and childhood HPP, clinical manifestations include defects in bone mineralization and bone deformities. Premature bony fusion of cranial sutures can lead to high intracranial pressure and cerebral damage^16^. In infantile HPP, the skeletal disease can lead eventually to respiratory complications^17,18^. Moreover, PLP accumulation is associated with convulsions in children because the phosphate group of this substrate prevents its translocation through the blood-brain barrier. This fact limit the bioavailability of PLP as a cofactor for many enzymatic reactions, such as the synthesis of neurotransmitters^19^. Premature loss of deciduous teeth is typical in childhood HPP^20^.

Due to the characteristic hypomineralization of this disease, HPP is often misdiagnosed as osteoporosis and treated with antiresorptive drugs, such as bisphosphonates. These drugs worsen the prognosis of HPP. For this reason, implementing a protocol in clinical practice to prevent the underdiagnosis of HPP and to treat adequately this disease is essential. In this context, the aim of this study was to assess the persistent low serum ALP levels, excluding secondary HPP causes due to another disorders^13,21^ or hypophophatasemia caused by some treatments such as glucocorticoids, chemotherapy or chlofibrate^22,23^, in order to design an useful protocol to detect positive cases of underdiagnosed HPP.

## Results

### Biochemical and clinical features in adults

The database of the Clinical Analysis Unit of the University Hospital San Cecilio of Granada recorded ~130,000 ALP analyses corresponding to 78,590 subjects (76,083 adults and 2,507 children) between the 1st of January and the 31st of December, 2016.

**Figure 1:**
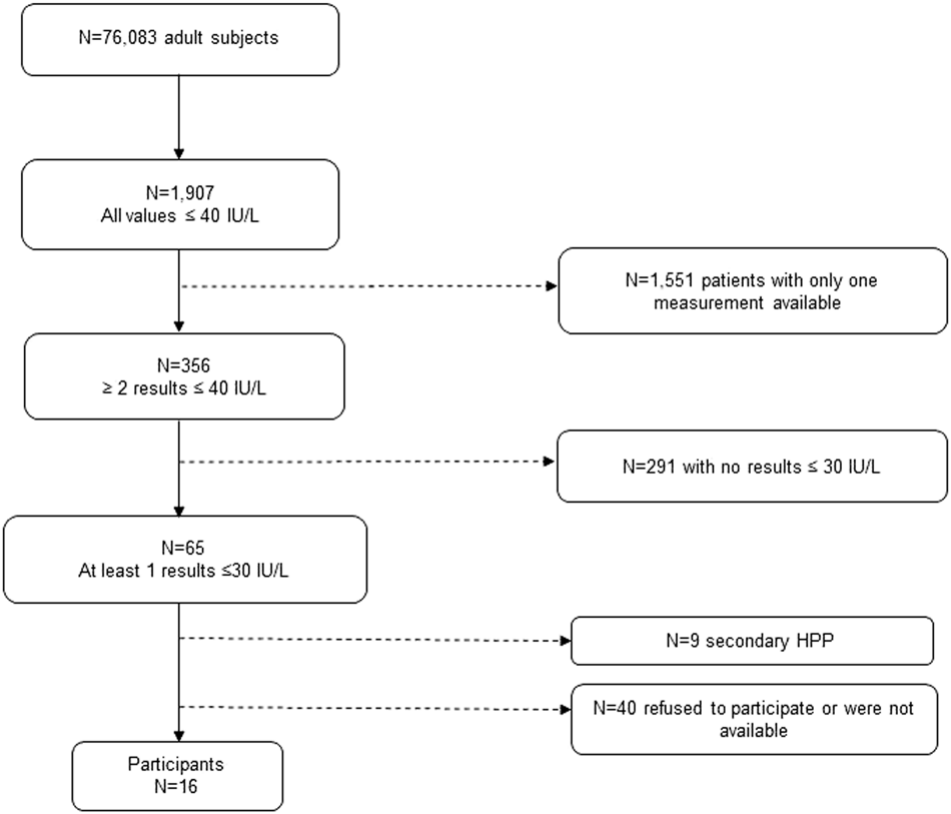
Flow chart of the recruitment of potential HPP patients from an adult population.

Among the adult population, we identified 1,907 subjects with ALP levels below the lower limit of the reference range (40 U/L). To reduce false positives, 1,551 subjects having only one ALP assessment were excluded. Among the remaining 365 subjects showing two or more ALP assessment ≤ 40 U/L, 65 subjects with at least one ALP assessment ≤ 30 U/L were selected. After reviewing the clinical records, 9 subjects were excluded because of secondary HPP causes (multiple myeloma (n = 1), milk-alkali syndrome (n = 1), malnutrition (n = 1) or because of death (n = 6)). Among the remaining 56 subjects, 16 subjects (50 ± 16 years, 81% women) agreed to participate in the study (Fig. 1).

Most of the 16 subjects were asymptomatic for HPP or had mild symptoms. None of them had been previously diagnosed with HPP. The main diagnosis of each patient is described in Table 1, including some diagnoses related to HPP, such as osteoarthritis (n = 2), inflammatory rheumatic disease (n = 1), osteoporosis (n = 3) and fibromyalgia (n = 1). However, most of the 16 subjects presented with other diagnoses non-related to HPP. None of them had a family history of HPP or rickets in childhood. None of them has been treated with bisphosphonates. Eight out of the 16 patients (7 of them females) had prevalent fractures (metatarsus, humerus, tibia, ankle, wrist, fibula or hip). Six out of the 16 patients (5 females and 1 male) had symptomatic chondrocalcinosis. Five out of the 16 patients reported dental abnormalities. Regarding biochemical determinations, 10 adults showed decreased ALP and increased PLP levels compared to the reference values. Seven out of these 10 patients presented with ALPL gene mutations. Two of these identified mutations corresponded to new variants not previously described.

**Table 1:** Clinical features, ALP levels, PLP levels and genetic study in the adult population.

N°	Age	Sex	History of fractures	Chondrocalcinosis	Dental abnormalities	Main Diagnosis	ALP (U/L)	PLP (ng/mL)	Genetic study
1	50	F	1 (metatarsal)	No	No	Multiple sclerosis	25	**58**.76*	**c**.**659G > T; p**.**Gly220Val**
2	53	M	No	No	No	Polycythemia vera, atrial fibrillation	30	13.95	(−)
3	69	F	1 (wrist)	No	No	Osteoarthritis	17	**55**.74*	**c**.**1366G > A; p**.**Gly456Arg**
4	27	F	No	No	Yes	Thiemann’s disease, ulcerative colitis	25	12.17	(−)
5	53	M	2 (metatarsal, ankle)	No	No	Dermatitis	26	**22**.99*	**c**.**473-2A > G**
6	43	F	No	Yes	No	Myasthenia gravis, hyperthyroidism	25	**23**.90*	(−)
7	48	F	1 (ankle)	Yes	No	Systemic sclerosis, osteoporosis	29	8.98	(−)
8	45	F	No	Yes	No	Lupus, colon cancer	30	12.02	(−)
9	34	F	3 (metatarsal, wrist, hip)	No	No	Idiopathic thrombocytopenic purpura	19	**24**.12*	**c**.**558G > A; p**.**Trp186**^**a**^
10	45	F	2 (tibia, fibula)	No	Yes	Ankle fracture	30	9.41	(−)
11	60	M	No	Yes	No	Acute pancreatitis, granular lymphocytic leukemia, cholelithiasis	20	**19**.66*	(−)
12	38	F	2 (metatarsal, ankle)	Yes	Yes	Ulcerative colitis	28	**37**.41*	(−)
13	30	F	No	No	Yes	Ankylosing spondylitis	22	**19**.48*	**c**.**558G > A; p**.**Trp186**^**a**^
14	65	F	No	Yes	Yes	Fibromyalgia, osteoarthritis, osteoporosis	18	**62**.26*	**c**.**1327G > A; p**. **Ala443Thr**^**b**^
15	70	F	1 (humerus)	No	No	Osteoporosis, breast cancer	**21**	**37**.25*	**c**.**1327G > A; p**. **Ala443Thr**^**b**^
16	36	F	No	No	No	Chronic inflammatory arthritis, fibromyalgia, lupus	26	9.47	(−)

^*^PLP levels higher than interval reference values. ^a^First genetic variant found. ^**b**^Second genetic variant found. (−) denotes a negative result.

### Genetic results in adults

Five out of 7 patients showing a positive genetic test (i.e., a mutation in the ALPL gene) presented with a history of fractures, one of them presented with chondrocalcinosis, and two of them reported dental abnormalities (Table 1).

**Figure 2:**
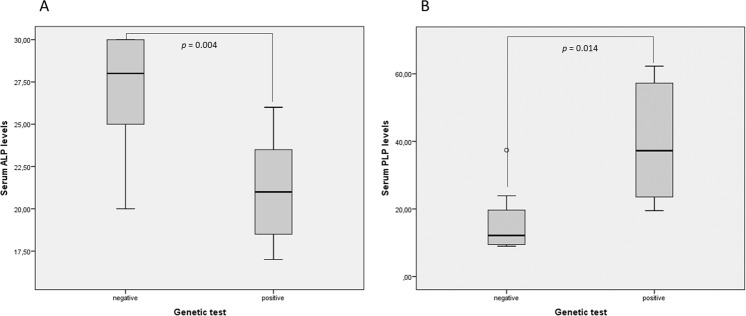
Serum ALP and PLP levels in the presence or absence of mutations in the ALPL gene. Panel A shows serum ALP levels and panel B shows serum PLP levels.

**Figure 3:**
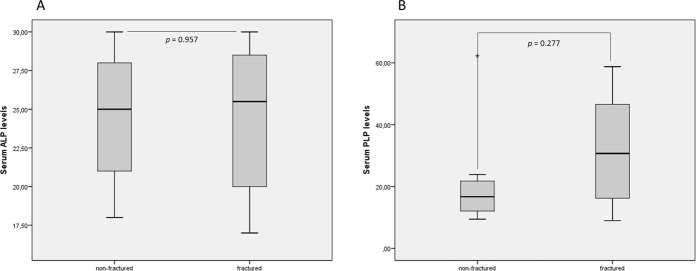
Serum ALP and PLP levels in the presence or absence of fractures. Panel A shows serum ALP levels and panel B shows serum PLP levels.

Among the subjects studied, we found that patients with a positive genetic test showed significantly lower levels of ALP and higher levels of PLP (Fig. 2). In addition, patients with fractures showed higher levels of PLP than those patients without fractures, although without reaching statistical significance (Fig. 3).

Two patients (patients 9 and 13, Table 1) presented with an ALPL mutation in exon 6 in heterozygosis, c.558G > A; p.Trp186*. Whereas another two patients (patients 14 and 15, Table 1) presented with an ALPL mutation in exon 12 in heterozygosis, c.1327G > A; p. Ala443Thr. These two mutations are new genetic variants, not previously described. The other three patients showed previously described ALPL mutations. One of them (patient 5, Table 1), presented with a mutation in exon 6 in heterozygosis, in the splicing canonical acceptor region (c.473-2A > G), leading an alteration in mRNA processing^24^. Another subject (patient 1, Table 1) presented with a mutation in exon 7 in heterozygosis (c.659G > T; p.Gly220Val^25^). The remaining subject (patient 3, Table 1) presented with a mutation in exon 12 in heterozygosis (c.1366G > A; p.Gly456Arg) finding a similar variant in previous studies^26^.

### Genetic results, biochemical and clinical features in the pediatric population

**Figure 4:**
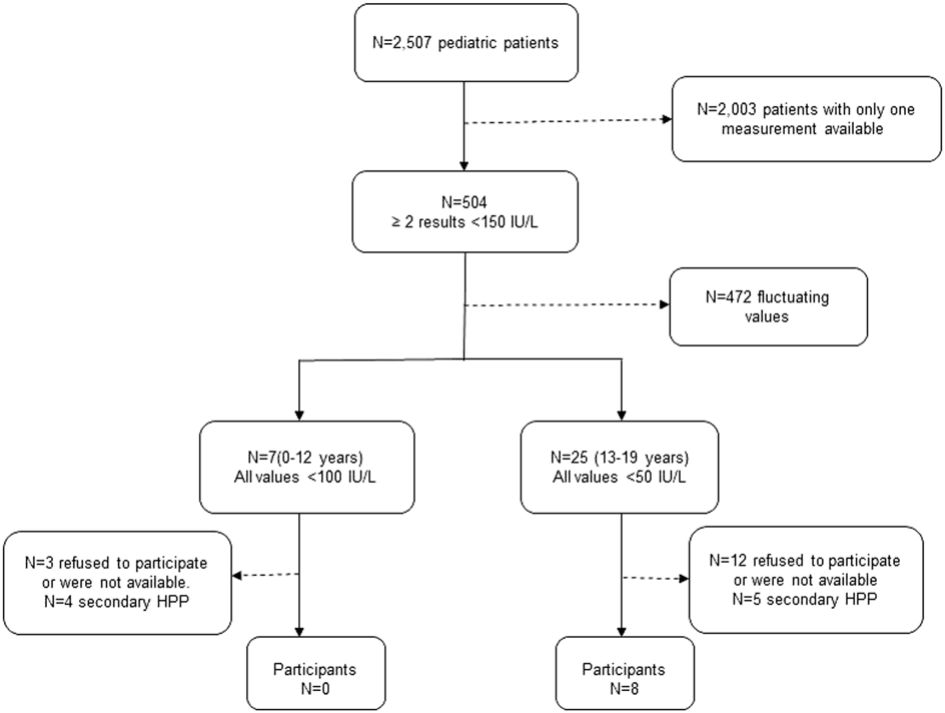
Flow chart of the recruitment of potential HPP patients from a pediatric population.

In the pediatric population, 2,003 out of 2,507 subjects with only one ALP assessment performed were excluded and 504 subjects showing ALP levels below 150 U/L were selected. Among the latter, those subjects presenting with some ALP assessment with serum levels above the reference limits were excluded (472 subjects). At this point, we classified the pediatric population into two groups based on age and ALP reference values (from 0 to 12 years and from 13 to 19 years). Therefore, 7 and 25 subjects with at least one determination below the threshold of 100 U/L or 50 U/L, respectively, were selected. After reviewing the clinical records, 4 children belonging to the first group and 5 from the second one, were excluded because of having secondary HPP causes [hypothyroidism (n = 3), malnutrition (n = 4) and leukemia or undergoing chemotherapy (n = 2)]. Finally, only 8 subjects belonging to the group from 13 to 19 years (18 ± 1 years, 87.5% females) accepted to participate in the study (Fig. 4). None of them had a family history of HPP or rickets. One of these 8 subjects had a history of fractures (femur, tibia, fibula and radius) by polytrauma. Only one of the 8 subjects had a diagnosis related to HPP (arthralgia), presenting most of them with other pathologies non-related to HPP (Table 2). Despite 2 out of the 8 subjects presented with low levels of ALP and a high level of PLP compared to the reference intervals, no mutations in the ALPL gene were found for none of them.

**Table 2:** Clinical features, ALP levels, PLP levels and genetic study in the pediatric population.

N°	Age	Sex	History of fractures	Chondrocalcinosis	Dental abnormalities	Main Diagnosis	ALP (U/L)	PLP (ng/mL)	Genetic study
1	18	F	No	No	No	Hearing loss	37	11.25	(−)
2	20	F	No	No	No	Multiple sclerosis	50	9.72	(−)
3	20	F	No	No	No	Lupus	40	10.05	(−)
4	19	F	No	No	No	Migraine	49	**32**.68*	(−)
5	20	F	No	No	No	Arthralgia	42	**23**.92*	(−)
6	19	M	4 (femur, tibia, fibula, radius)	No	No	Polytrauma	46	11.98	(−)
7	20	F	No	No	No	Asthma	40	9.77	(−)
8	19	F	No	No	No	Gastrointestinal infection	43	18.43	(−)

^*^PLP levels higher than interval reference values. (−) denotes a negative result.

### Functional validation of two new mutations using a cell-based assay

**Figure 5:**
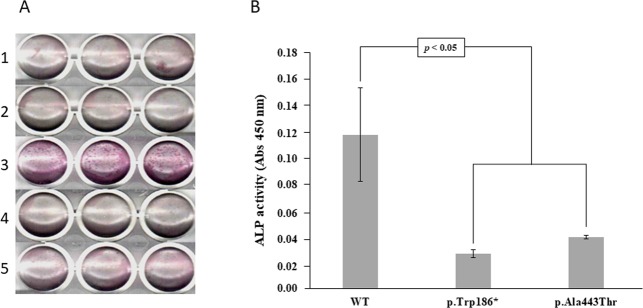
Cell based-assay to determine the ALP activity in transfected and non-transfected HEK293T cells. Panel A shows the qualitative results of ALP activity in HEK293T cells in the following conditions: non-transfected cells, cells transfected with pCDNA 3.1, cells transfected with pCDNA 3.1-ALPL WT, cells transfected with pCDNA 3.1-ALPL c.558G > A mutant (p.Trp168*), cells transfected with pCDNA 3.1-ALPL c.1327G > A mutant (p.Ala443Thr) in lines 1, 2, 3, 4 and 5 respectively. Panel B shows the quantitative results of the ALP assay expressed as absorbance at 450 nm. The results are expressed as means and standard error, derived from three independent experiments.

The ALP activity was measured in HEK293T cells transfected with plasmids containing the wild-type (WT) ALPL or the ALPL mutants. The truncated mutant (p.Trp186*) showed a dramatic ALP activity decrease comparable to both, the non-transfected cells or the cells transfected with empty vector (Fig. 5). The second mutant (p.Ala443Thr) also showed a significant reduction in ALP activity, although the ALP activity was higher than in the truncated mutant, suggesting that the second mutation has a lower impact in TNSALP 3D structure.

The transfection and expression studies performed to verify the validity of the experiment have demonstrated that the ALPL transcriptional expression was similar in cells transfected with plasmids containing the WT or the ALPL mutants. A higher expression of ALPL gene was found in WT ALPL cells or ALPL mutant cells compared to cells transfected with the empty vector. The empty vector was used as a negative control to assess the contribution of endogenous expression of ALPL gene in HEK293T cells (Fig. S1).

### Effect of new mutations based on a 3D model of the TNSALP structure

**Figure 6:**
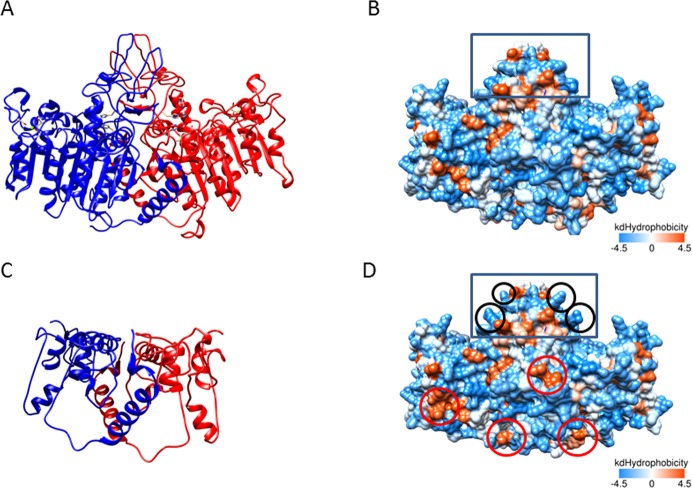
3D modeling of WT and both new mutants of TNSALP. The structure modeling is based on the sequence homology between TNSALP and the placental isozyme (PDB ID: 1E2W). Panels A and C show the 3D structure as a ribbon representation of WT TNSALP and the truncated mutant, respectively. The two monomers of the protein are colored in red and blue. Panels B and D show the 3D structure as a hydrophobic surface representation of WT TNSALP and the second mutant (p.Ala443Thr). The color of the molecular surface by amino acid hydrophobicity has been used as follows: from dodger blue for the most hydrophilic residues, changing to white, to orange red for the most hydrophobic residues. The most hydrophilic changes introduced in the mutant compared to the WT are delimited with black circles and the most hydrophobic changes, with red circles. The crown domain is delimited with a rectangle.

In order to predict the effect of these two newly identified mutations on the TNSALP structure, a 3D model based on the sequence homology between TNSALP and the placental isozyme was obtained using the web-server SWISS MODEL. Although both protein sequences share only a 57% of identity^27^, the structure of both molecules is highly conserved (Fig. S2). The first new mutation identified (c.558G > A; p.Trp186*) produces a truncated TNSALP protein which conserves only 1/3 of its sequence, affecting this to the loss of the main part of its structure (Fig. 6A,C). Bioinformatics analysis of the second new mutation identified (c.1327G > A; p. Ala443Thr) revealed that even maintaining a very similar backbone 3D structure, the p.Ala443Thr mutant showed relevant modifications in the general polarity of the whole molecule, increasing the hydrophobic surface clusters (Fig. 6B,D) which may affects to its function.

## Discussion

Our study shows that HPP is an underdiagnosed disease. An appropriate protocol to detect HPP in a clinical setting in tertiary care hospitals is required.

We found that 0.12% of the subjects undergoing ALP assessments in 2016 at the University Hospital San Cecilio of Granada had persistently low serum ALP levels, although not all subjects were affected by HPP. This disorder is often overlooked in clinical practice because low serum ALP levels do not capture the attention of clinicians, in contrast to the finding of elevated serum ALP levels. This is a current problem limiting the HPP diagnosis. The finding of low serum ALP level is not synonymous of suffering HPP. Accordingly, a recent study showed that only half of the patients between 20 and 77 years old presenting with unexplained low ALP levels had a mutation in the ALPL gene^24^. Furthermore, some clinical conditions can also be associated with a reduction or increase in the levels of circulating ALP (such as pregnancy, several hepatobiliary diseases, orthopedic surgery and/or recent fractures)^28–30^, which could mask the diagnosis of HPP. All these conditions must be considered to interpret properly any altered level of total ALP. Therefore, our results showed a decrease in serum ALP levels and an increase in serum PLP levels in patients presenting with some mutation in ALPL gene. However, this relation does not occur between serum ALP levels and the presence of fractures. We found increased serum PLP levels close to significance in patients with fractures. In this context, an accumulation of natural substrates for TNSALP and clinical and radiographic findings should be considered, in conjunction with low total serum ALP level, in order to establish a correct diagnosis of HPP.

Our study found 7 out of 16 adult patients with a positive genetic test for HPP. In consistency with the proportion reported by McKiernan study^30^, we found 5 subjects (4 females and 1 male) that presented with all the clinical hallmarks of HPP (low total serum ALP level, high PLP levels and a history of prevalent fractures) (patients 1,2,5,9 and 15, Table 1). In addition, we identified two females with positive genetic test, low serum ALP level and high PLP levels^31^ but without history of prevalent fractures (patients 13 and 14, Table 1). However, both of these patients showed dental abnormalities, one of them presenting with chondrocalcinosis too. This symptomatology could fit with adult HPP or odonto-HPP^5^.

Additionally, two patients (both females) presented with serum ALP and PLP levels consistent with HPP diagnosis. However, despite the genetic test was negative, both patients could be affected by HPP, since the biochemical and clinical manifestations were associated with common HPP-related symptoms (patients 11 and 12, Table 1). This inconsistency could be explained by additional factors that can influence the clinical expression of HPP. The negative results in the genetic test could be explained because there could be some proteins or transcription factors indirectly related to TNSALP regulation, such as transcription factor RUNX2, leading to the characteristic low serum levels of ALP found in HPP in the absence of the mutation in the ALPL gene^32^. Moreover, the regulation of extracellular PPi levels, responsible for some HPP symptoms, is complex and it involves several genes in addition to ALPL^33^. Furthermore, mutations in some non-coding regions, such as promoter or intergenic regions of the ALPL gene, which are undetected by conventional sequencing could explain the negative results of the genetic test similarly to previously reported results of other studies^34^.

Patients 9 and 13 (Table 1) presented with a novel mutation (c.558G > A; p.Trp186*) leading to an amino acid change, generating a shorter version of the TNSALP protein by introducing a stop codon at position 186. The functional validation using a cell-based assay performed for this mutation showed a dramatic decrease in the ALP activity (Fig. 5). This high decrease could be explained because this mutation generates a TNSALP truncated version that has missed almost 2/3 of its sequence (Fig. 6A,C). This fact implies that the truncated protein has lost some important structural determinants, such as an active site at the 110 position, some metal binding sites and the cysteine 139 involved in disulfide bond formation^35^. Some structural studies confirm that the metal in the metal-binding site is a calcium ion, which could play a critical role in the TNSALP function^27^. Mutations affecting these structural determinants could explain the drastic loss of activity in this protein.

However, the clinical phenotype associated with the c.558G > A; p.Trp186* mutation is not specific, since one of these patients showed fractures at several locations and the second patient presented with dental abnormalities. This difficulty in linking genotype with phenotype can be explained by the influence of other genetic, epigenetic or non-genetic factors in this disorder^36^. The symptomatology associated with this genetic variant could correspond to adult HPP.

Patients 14 and 15 (Table 1), showed another novel ALPL mutation involving an amino acid replacement at the position 443 of the protein (c.1327G > A; p. Ala443Thr). The results of site-directed mutagenesis performed to validate this mutation showed a strong reduction in the ALP activity (Fig. 5). The replaced amino acid is located at 443 position, next to the crown domain of the protein^27^ (Fig. 6B,D). This domain contains the collagen binding site, which has been identified as important for the TNSALP function^37^ and also is involved in the monomer-monomer interaction^27^. This mutation implies the appearance of several hydrophobic clusters, all over the dimer surface (Fig. 6B,D). This alteration could affect the dimerization of protein, and collagen binding. Both processes are essential to the TNSALP function and could explain the decreased ALP activity found in the *in vitro* assays. Surprisingly, this mutation affected significantly the general hydrophobicity of the protein, while keeping the secondary, tertiary and quaternary structures almost identical (Fig. 6B,D). Although this mutation had not been previously described, two different variants at the same position has been reported by other groups (p.Ala443Val^26^ and p.Ala443Glu^7^), showing both variants symptomatology related to adult HPP. In addition, mutations located at crown domain have been related to severe or moderate HPP depending on location and homozygous or heterozygous genotype, reinforcing the important role of this domain in the ALP activity^27^.

In our study, the clinical features of this mutation seem to be associated with a low bone mineral density in both affected patients, who have been diagnosed with osteoporosis.

Mild symptoms associated to these novel mutations could be explained by the heterozygosity of both mutations. Both new genetic variants have been annotated in The Tissue Nonspecific Alkaline Phosphatase Gene Mutations Database on the 27 of October, 2018^2^.

Patients 1, 3 and 5 (Table 1) showed the above described ALPL gene mutations and all of them seem to be related to adult HPP, since all affected patients reported a history of fractures at several locations (Table 1). In previous studies, the mutation found in patient 3, which leads to an amino acid replacement in position 456 was associated with adult HPP with mild symptoms^26^. Likewise, the mutation found in patient 1 (Table 1) was described in odonto-HPP form by Taillander *et al*.^25^. Mutation in patient 5 (Table 1) was found in an intergenic region which affects mRNA processing. This mutation was associated with an asymptomatic HPP form and with very mild form in other studies^24^. In consistency with this observation, we found two fractures (in metatarsus and ankle) in the affected patient, with no additional symptoms related to HPP. Based on these results, all the genetic mutations found in our study subjects seem to be related with adult HPP with very mild symptoms.

The bioinformatics pathogenic prediction tools suggest that all the mutations found in our study, including the two new genetic variants identified, are pathogenic according to the classification criteria of the American College of Medical Genetics and Genomics (ACMG)^38^. According to Varsome database classification^39^, the first novel mutation, (c.558G > A; p.Trp186*) found in patients 9 and 13 (Table 1), is classified as pathogenic (class 5), whereas the second mutation, (c.1327G > A; p. Ala443Thr), found in patients 14 and 15 (Table 1), is classified as likely pathogenic (class 4).

Regarding the pediatric population, we did not find any patient with positive results in the genetic test. In addition, none of the 8 children evaluated presented with chondrocalcinosis or dental abnormalities. Only one of them had a history of prevalent fractures in several locations (patient 6, Table 2), although this patient did not present with PLP levels higher than the reference interval. However, two children, both females (patients 4 and 5, Table 2) were found with ALP and PLP levels consistent with the common reference intervals for HPP. Despite no history of prevalent fractures nor dental abnormalities were found for these two children, abnormal ALP or PLP levels should be considered for further studies to evaluate the presence of a likely asymptomatic or moderate HPP in the absence of genetic mutations, as argued above.

Similarly to other studies^30,40^, we found a higher proportion of females (n = 6, 86% approximately) than males (n = 1, 14% approximately) diagnosed with HPP (with positive genetic test) in our final adult cohort. Despite no significant sex differences were found in our initial adult cohort with persistent low serum levels of ALP (54% females, 46% males), we cannot affirm that HPP is more prevalent in females than in males in our study population. This is because most subjects with low ALP levels refusing to participate in our study or that were not available were males, which led to a final cohort with higher proportion of females than males (81% females *vs* 19% males approximately, n = 16). The higher prevalence of females among subjects affected by HPP found in the present study and in other studies could be explained because bone-related disorders are found more frequently in women (such as postmenopausal osteoporosis), which finally are affected by HPP. In addition, more awareness about bone-health is found in women than in men, which leads to a higher participation of females than males in this type of study.

In summary, we can conclude that HPP is an underdiagnosed disease showing a higher proportion of affected patients in a tertiary care hospital than previously estimated. Following the protocol described in the present work, we found 7 affected adult patients, without previous diagnosis of HPP, out of the 16 subjects studied. This data indicates that almost half of the population studied (~44%) was not well identified in routine clinical practice. If we extrapolate this data to the current population of Spain, this could mean 4,000 potential cases of HPP currently not diagnosed. Considering the proportion of potential HPP patients who did not participate in this study for different causes (71%, Fig. 1), and according to our results, the current estimate of potential undiagnosed HPP cases in Spain could reach up to 15,000 cases. This implies that the estimated prevalence of mild HPP in our country would be double than the previously published for Europe^1^ (1/3,100 *vs* 1/6,370). Among the 7 subjects affected by HPP, we found two new ALPL mutations not previously described. Both mutations have been annotated in The Tissue Nonspecific Alkaline Phosphatase Gene Mutations Database. Both genetic variants are related to the adult HPP clinical form.

In addition, we found 4 more patients with negative results for the genetic test (2 adults and 2 children) but presenting with the typical HPP symptomatology. Further studies are required to verify if these subjects are also affected by HPP.

Because of its clinical features, HPP could be wrongly mistaken for osteoporosis or other bone-related diseases. The treatment of these patients with antiresorptive drugs is frequent in tertiary care hospitals, leading to the worsening of their prognosis. Therefore, it is important to establish a correct clinical assessment to perform an adequate diagnosis of this disorder, and therefore, to provide the appropriate management of the affected patients.

## Methods

### Study population

A total of 78,590 subjects were included in this retrospective study. These subjects had undergone a previous ALP assessment between the 1st of January and the 31st of December, 2016. The subjects included were evaluated at the Clinical Analysis Unit of the University Hospital San Cecilio of Granada. The database was divided into adult (76,083 subjects) and pediatric population (2,507 subjects).

Among the adult population, subjects who showed ALP levels below the lower limit of the reference range (40 U/L) in at least two assessments were recruited. To increase the specificity, those selected subjects presenting ALP levels ≤ 30 U/L in at least one assessment were recruited. Among the pediatric population, subjects who showed ALP levels below 150 U/L in at least two assessments were recruited. Selected subjects were classified into two groups based on age and ALP reference values: a threshold of 100 U/L was chosen for the group with ages from 0 to 12 years old and a threshold of 50 U/L was chosen for the group with ages from 13 to 19 years old. Pediatric subjects with ALP levels below these thresholds were included in the present study.

The clinical records of selected subjects were reviewed to exclude those subjects with low ALP levels caused by secondary HPP^13,21^. Subjects selected to participate in the study were contacted to sign an informed consent. In the case of subjects under the age of 18 years, informed consent was signed by a parent and/or legal guardian for study participation. An individualized and personal interview about potentially related HPP symptoms was conducted to perform a structured clinical evaluation.

Two venous blood samples were collected from each participant at the Clinical Analysis Unit for PLP determination and for the genetic study.

The present study was approved by the ethics committee of the University Hospital San Cecilio of Granada in accordance with the principles of the World Medical Association Declaration of Helsinki (Project ID: 0768-N-17. Research Ethics Committee of Granada Center (CEI-Granada) on 5 September 2017).

### Biochemical analyses

The ALP levels were measured in blood samples by a colorimetric method in an AU5800 analyzer (Beckman Coulter, California, EEUU). The method based on the recommendations of the “International Federation for Clinical Chemistry” (IFCC) was used for this purpose. The ALP activity was determined by measuring the rate of conversion of p-nitrophenyl phosphate (pNPP) to p-nitrophenol (pNP) in the presence of magnesium and zinc ions and of 2-amino-2-methyl-1-propanol (AMP) as a phosphate acceptor at pH 10.4. The rate of change in absorbance due to the formation of pNP was measured bichromatically at 410/480 nm and this rate is a direct function of the ALP activity in the sample (reference range in adult population: 40–120 U/L). The reference ranges for the pediatric population are shown in Table 3. According to these values, we established 100 U/L as threshold for the pediatric group from 0 to 12 years old and 50 U/L for the pediatric group from 13 to 19 years old to design our protocol.

**Table 3:** Reference ALP values in the pediatric population based on age and sex.

Pediatric population	Male (U/L)	Female (U/L)
1–30 days	75–316	48–406
30 days–1 year	82–383	124–341
1–3 years	104–345	108–317
4–6 years	93–309	96–297
7–9 years	86–315	69–325
10–12 years	42–362	51–332
13–15 years	74–390	50–162
16–18 years	52–171	47–119

Plasma PLP concentrations, as an indicator of vitamin B6 adequacy, were determined by high performance liquid chromatography (HPLC) at the Clinical Analysis Unit of University Children Hospital Niño Jesús (Madrid). The chromatographic measurement was performed on an isocratic HPLC system with a fluorescence detector. The excitation and emission wavelengths for the detector were 320 nm and 415 nm, respectively. The reference range was 6.4–18.5 ng/mL.

### Sequencing of ALPL gene

Extraction of DNA from peripheral blood lymphocytes was performed. Amplification was performed by PCR. Subsequent sequencing of the coding regions and exon-intron junctions of the ALPL gene was carried out using as reference the truncated sequence NM_000478.4 at the INGEMM (Instituto de Genética Médica y Molecular) of the University Children Hospital Niño Jesús (Madrid), according to the methodology described by Riancho *et al*., 2016^24^.

### Transfection studies

The pcDNA3.1+ vector containing the full-length wild type (WT) ALPL gene was obtained from GenScript company (Clone ID: OHu23066). The two new mutations identified in the present study were obtained by site-directed mutagenesis and they were introduced in OHu23066 clone using the HindIII and BamHI restriction sites. The mutated cDNAs were fully sequenced to demonstrate that the target mutation was inserted. Mutated and WT plasmids were transiently transfected into HEK293T cells for 48 hours. Transfections were performed by the lipofection method with LipoD293 DNA *In Vitro* Transfection Reagent (SignaGen Laboratories) following the manufacturer’s instructions. RT-qPCR experiments were performed to control transfection and ALPL exogenous expression. The total RNA was isolated from each transfected culture and from the non-transfected control using a manual homogenizer and Trizol Reagent (Ambion). RNA was treated with DNAse (Qiagen), then, cDNA was synthesized from 600 ng of RNA using the iScript cDNA synthesis kit (BioRad) following the manufacturers’ instructions. Quantitative PCR was performed using the PowerUp SYBR Green Master Mix (Thermo Fisher Scientific) in a CFX96 Real Time thermocycler (BioRad). The set of primers designed to amplify a 121 bp fragment of the ALPL gene from both WT and mutant plasmids are the following: ALPL-F: 5′-TGGCACCTGCCTTACTAACT-3′ and ALPL-R: 5′-CACGTTGGTGTTGAGCTTCT-3′. Gene expression data were normalized to the expression of the reference gene Ribosomal Protein L13 (RPL13) and reported as normalized ALPL expression. The following set of primers was used to amplify the reference gene: RPL13-F: 5′-CGTAAGATCCGCAGACGTAAGGC-3′ and RPL13-R: 5′-GGACTTGTTCCGCCTCCTCGGAT-3′.

### ALP activity in transfected cells

The ALP activity was determined using Alkaline Phosphatase Detection Kit (Merck Millipore) following the methodology recommended by the manufacturer. The enzyme activity of ALP was measured with a spectrophotometer (Dynex Technologies) at 450 nm to detect the chromogenic product as a result of the ALP activity.

The pcDNA3.1 vector contains a CMV promoter. Consequently, the endogenous ALP activity in HEK293T cells has a low contribution to the total ALP activity in transfected cells. In order to assess the actual contribution of these mutants to ALP activity, the endogenous ALP activity determined in HEK293T cells transfected with pcDNA3.1 was subtracted to the results of the ALP activity determined in HEK293T cells transfected with pcDNA3.1 containing WT or ALPL mutants. The results were expressed as ALP activity based on absorbance at 450 nm measured in each condition. The results are represented as means with standard error, derived from three independent experiments.

### 3D structural modeling

To check the effect of both new mutations identified in this study, the 3D modeling of WT and TNSALP mutants was completed using SWISS MODEL (https://swissmodel.expasy.org/). The structure modeling is based on the sequence homology between TNSALP and the placental isozyme (PDB ID: 1EW2). The new pdb files for WT, p.Trp168* and p.Ala443Thr TNSALP are available upon request. Ribbon representations and hydrophobic surface representations were obtained using UCSF Chimera software^41^. Mutation-related residues in the present study were positioned using the open source https://swissmodel.expasy.org/repository/uniprot//P05186.^35^.

### Statistical analyses

Kolmogorov-Smirnov test was used to assess the normality of distribution of continuous variables. Comparisons of continuous variables among groups were performed using unpaired Student’s *t* test. The ALP measure was calculated based on absorbance at 450 nm in three independent experiments in transfected cells, and Shapiro-Wilk test was used to study the distribution of this variable. Log 10 transformation was performed to normalize these data and ANOVA was performed to compare the differences between groups. Statistical significance was set at *p* < 0.05 (two-tailed). Statistical analysis was performed with specific software, SPSS version 22.0.

## Supplementary information

Supplementary Information
