# Supplementary material for: Epidemiological, Clinical and Genetic Study of Hypophosphatasia in A Spanish Population: Identification of Two Novel Mutations in The Alpl Gene
Source: Sci Rep. 2019 Jul 2;9:9569. doi: 10.1038/s41598-019-46004-2 (PMC6606844; doi:10.1038/s41598-019-46004-2)

SUPPLEMENTARY INFORMATION

EPIDEMIOLOGICAL, CLINICAL AND GENETIC STUDY OF HYPOPHOSPHATASIA IN A SPANISH POPULATION: IDENTIFICATION OF TWO NOVEL MUTATIONS IN THE ALPL GENE

Cristina García-Fontana[1,2,+], Juan M Villa Suárez, [3,4,+], Francisco Andújar-Vera [1,2], Sheila González-Salvatierra [1,4], Gonzalo Martínez-Navajas [5,6] , Pedro J Real [5,6], José M Gómez Vida [7], Tomás de Haro Muñoz [3], Beatriz García-Fontana [1,8,*], Manuel Muñoz Torres [1,4,8,9].

[1]University Hospital San Cecilio. Instituto de Investigación Biosanitaria (Ibs.GRANADA). Granada, Spain

[2]Fundación para la Investigación Biosanitaria de Andalucía Oriental (FIBAO). Granada, Spain

[3]Clinical Analysis Unit. University Hospital San Cecilio. Granada, Spain

[4]Department of Medicine. University of Granada. Granada, Spain

[5]Gene Regulation, Stem Cells & Development Lab. GENYO. Centre for Genomics and Oncological Research: Pfizer-University of Granada-Andalusian Regional Government. Granada, Spain

[6] Department of Biochemistry and Molecular Biology I, University of Granada. Granada, Spain.

[7] Pediatric Unit. University Hospital San Cecilio. Granada, Spain

[8] CIBERFES. Instituto de Salud Carlos III. Granada, Spain

[9] Endocrinology and Nutrition Unit. . University Hospital San Cecilio. Granada, Spain

*Corresponding author: Beatriz García Fontana. Address: Avda. Madrid, 15, 1st floor, 18012, Granada, Spain. Phone: +34 958023460. e-mail: bgfontana@fibao.es

[+] The first two authors contributed equally to this work.

Figure S1. Normalized transcriptional expression of WT ALPL and both new identified mutants using RPL13 as reference gene. A control containing cDNA from cells transfected with pCDNA 3.1 was included. The results are expressed as means and standard error derived from three independent experiments.

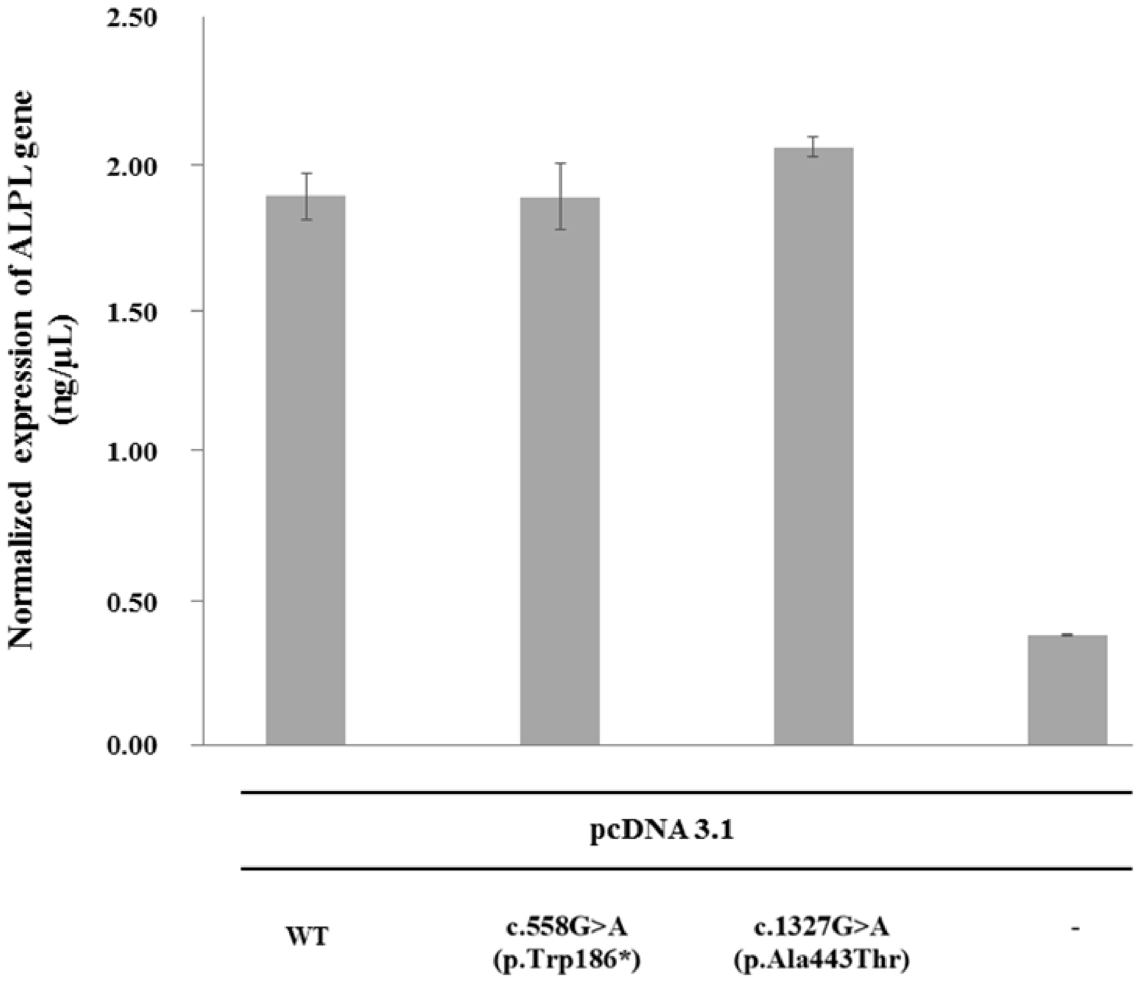

Figure S2. Alignment and 3D model visualization of TNSALP and placental isozyme isoform. Panel A shows the sequence alignment of TNSALP (first line) and placental isozyme (second line). Homologous residues are indicated in bold in the second line. The α-helices are delimited with a circle and β-strands are delimited with an arrow. Panel B shows the 3D modeling of TNSALP generated by SWISS-MODEL repository licensed under the CC BY-SA 4.0 Creative Commons Attribution-ShareAlike 4.0 International License (https://creativecommons.org/licenses/by-sa/4.0/legalcode). Panel C shows placental isoenzyme 3D structure (PDB ID: 1E2W). In both 3D models, both monomers of the protein are shown in red and blue.

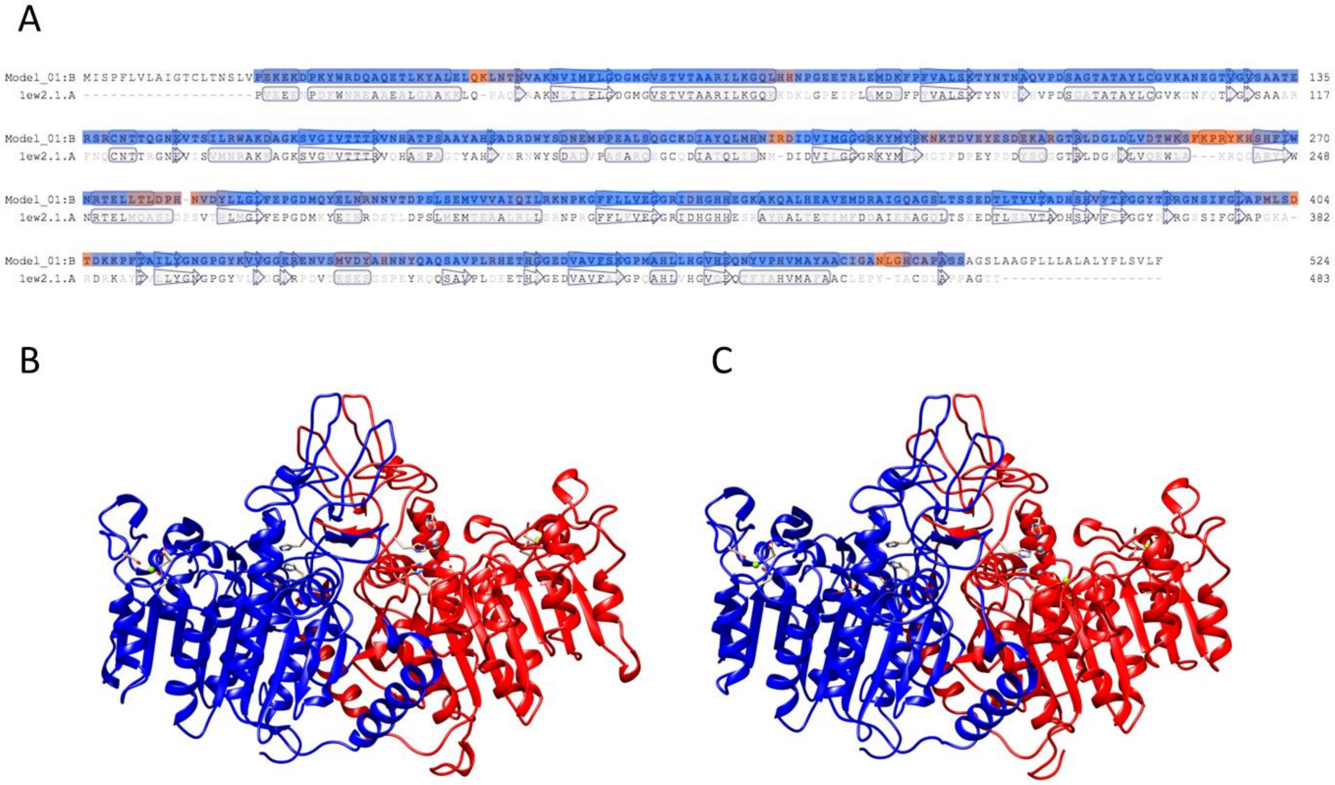

Supplement: Supplementary file 1 — Supplementary Information [file 41598_2019_46004_MOESM1_ESM.pdf]
